# Influence of an aluminium concentrator corrosion on the output characteristic of a photovoltaic system

**DOI:** 10.1038/s41598-020-78548-z

**Published:** 2020-12-14

**Authors:** Frederico Mota, João Paulo Neto Torres, Carlos A. Ferreira Fernandes, Ricardo A. Marques Lameirinhas

**Affiliations:** 1 Instituto Superior Técnico, Lisbon, 1049-001 Portugal; 2 Instituto de Telecomunicações, Lisbon, 1049-001 Portugal; 3 Academia Militar, Av. Conde Castro Guimarães, Amadora, 2720-113 Portugal

**Keywords:** Solar energy and photovoltaic technology, Energy efficiency, Sustainability

## Abstract

The climate changes observed over the last decades have been promoting a massive transformation on the energy sector, that is still, in truth highly dependent on fossil fuels. Renewable energies are a plausible alternative, because they have lower emissions of toxic gases in comparison with non-renewable ones. In the group of renewable energies, solar technology has the biggest overall potential, mainly because it is cheap and easy to set. Several solar technologies allow to equip their photovoltaic panels with concentrators, mostly to increase the output power and possibly their efficiency. However, some problems related to the use of concentrators have to be dealt in order to improve the entire photovoltaic system performance. One of these issues is the corrosion of the concentrators, leading to a premature ageing and, consequently an increase in maintenance costs. This problem is going to be analysed in this paper, presenting some simulation from a ray traicing software and also some experimental results, from our own laboratory experiences. The used software allows to trace the solar rays of the concentrator, in order to assess the effect of the defects caused by corrosion due to the ambient circumstances. After it, experimental results will help to analyse this effect and to prove simulation ones.

## Introduction

In the last decades, a tremendous increase on energy demand has provided a fantastic opportunity to the appearance and development of renewable technologies. Of all renewable technologies, the photovoltaic one has become quite important, because it is noiseless, it has no carbon dioxide emissions during operation, it is scalable, and it is characterised by a low prices per kWh, requiring simple operation and maintenance processes associated to easy set-ups, in comparison with non-renewable energy sources and technologies^1–15^. Even though we are a long way from replacing completely and unconditionally fossil fuel sources.

In terms of energy consumption, the actual world demand is approximately 10 terawatts (TW) per year^3,14^. However, it is expected that, by the 50s of XXI century, this value is going to significantly increase to 30 TW^3,14^.

Nowadays, new techniques and processes are being studied in order to increase and improve the way we can benefit from the solar radiation to generator electrical energy. In order to reach high efficiency values, new sets of cells, new formats and concentrators are being used^1–15^.

The main idea behind the use of a concentrator is to direct solar rays from the its surface to a receiver, that may be solar cells or fluids. Concentrators can decrease the number of expensive cells used per area without affecting its performance, leading to a decrease of price kWh generated. On the other hand, problems may arise while using these Concentrator-Photovoltaic (CPV) systems, mainly due to corrosion^3–18^.

The aim of this study is to acknowledge the effects that the degradation of concentrators, due to corrosion, can have in the output characteristic of a system composed by an aluminium concentrator and silicon solar cells. Firstly, a computer simulation is made to analyse how the optical errors of the corrosion in aluminium concentrators affect the output power of the system. Secondly, laboratory experiences are conducted to study the effects of aluminium corrosion on the system output characteristics.

## Background

### Solar spectrum

The energy emitted by the sun that is transmitted as electromagnetic radiation is called solar radiation and it can be characterised by its frequency, *f*, wavelength, $\lambda$, or photon energy, $E_f$. These properties are related by expressions (1) and (2), where ${c}_{0}$ is the *celeritas* symbol (light velocity in vacuum) and h is the Planck constant.

1 $$\begin{aligned}&{c}_{0} = \lambda f \end{aligned}$$

2 $$\begin{aligned}&E_f = hf \end{aligned}$$

**Figure 1:**
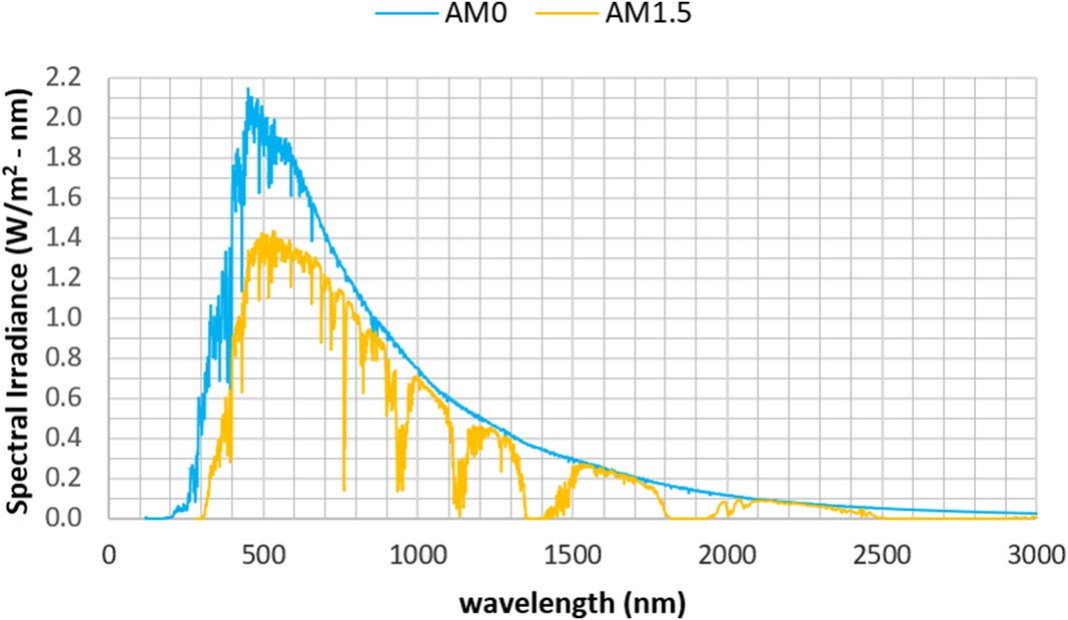
Solar radiation spectrum (Adapted from^19^).

“The sun provides more energy in four hours than the human race consumes in all forms in an entire year”^18^, however not all reach the Earth’s atmosphere. On top of that, after radiation pass through Earth’s atmosphere, its average intensity value is about 1000 W/m$^2$, but it oscillates, for example, with weather conditions. On Fig. 1 is presented the solar radiation spectrum, out of the atmosphere (AM 0) and the clear sky distribution (AM 1.5)^3–18^.

### Solar cells

Solar cells can convert incident radiation in electricity based on the photoelectric principle. Even though new technologies have been studied to improve the efficiency and adaptability of silicon cells, these are the most used today.

To characterise a solar cell, some specifications can be presented, as the short circuit current, $I_{SC}$, open circuit voltage, $V_{OC}$ and the maximum power point, defined by its power, $P_{mp}$, and its voltage and current, $V_{mp}$ and $I_{mp}$. These values have a huge dependence on the incident light intensity, which is modelled by a photovoltaic current, $I_{pv}$. Moreover, they depend on the cell material, which is described by the reverse bias saturation current of its junction, $I_{0}$ and its ideality factor, n. Thus, the equivalent expressions (3) and (4) are used to describe the voltage and current of a solar cell, where $V_T = \frac{kT}{q}$ is the thermal voltage, whereon T is the cell temperature, k the Boltzmann constant and q is the electron charge^1–18,20^.

3 $$\begin{aligned} V = n\,V_T\,\ln {\left( \frac{I_{pv}-I}{I_0}\right) } \end{aligned}$$

4 $$\begin{aligned}&I = I_{pv} - I_0\left( e^{{\frac{V}{nV_T}}}-1\right) \end{aligned}$$

**Figure 2:**
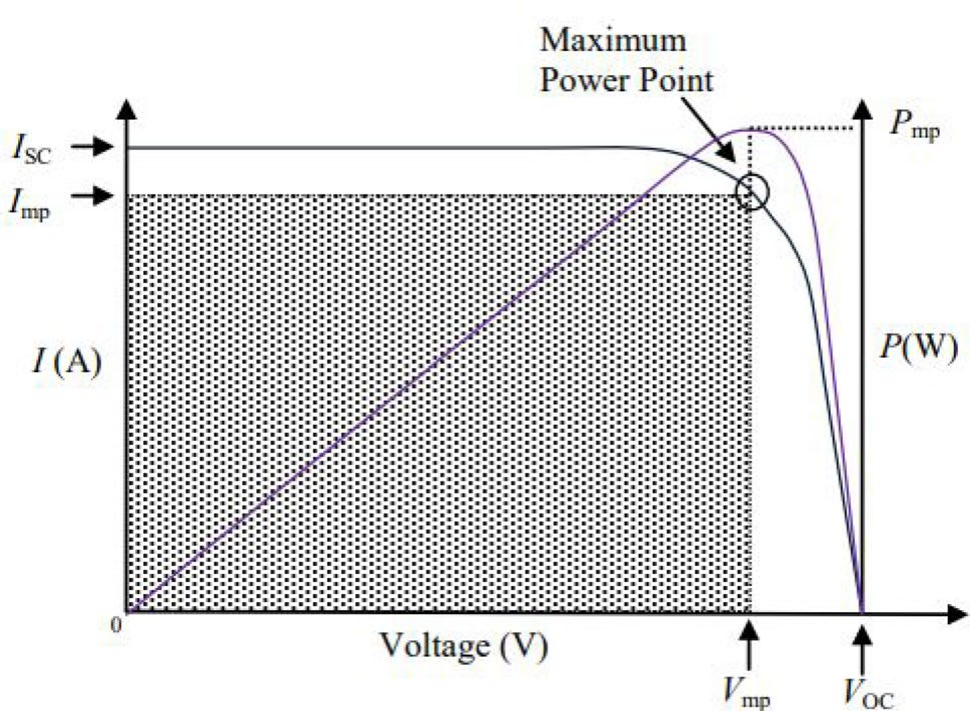
I(V) characteristic curve of a solar cell (Adapted from^11^).

Based on these expressions, it is possible to determined the I(V) characteristic of a certain cell. On Fig. 2 is illustrated not only a I(V) curve, but also a P(V) curve. There, the short circuit and the open circuit point are set, as well as the maximum power point.

Analysing expression (3) or (4), it is possible to reach expressions (5) and (6), for the short circuit and open circuit points.

5 $$\begin{aligned} V_{OC} = \frac{n\,k\,T}{q}\ln {\left( \frac{I_{pv}}{I_0}+1\right) } \end{aligned}$$

6 $$\begin{aligned}&I_{SC} \approx I_{pv} \end{aligned}$$

In order to increase the output power, it is possible to associate several solar cells. If $N_s$ identical cells are in series, then ideally the current should be the same and their total voltage should be $N_s$ times higher than the one for a single cell. On the other hand, if $N_p$ identical cells are in parallel, then ideally the voltage should be the same and their total current should be $N_p$ times higher than the one for a single cell. Thus, any other combination of solar cells, for example a photovoltaic (PV) module or panel, can have their open circuit, short circuit and maximum power points calculated, analysing the series and parallel associations.

### Solar concentrators and their corrosion degradation

Solar concentrators are being used in many solar applications, for example to heat fluids, in this case actuating as thermal collectors, or to increase the output power of a certain PV panel, without increase its active area^1–15,20,21^.

Generally, concentrators are aluminium made because of its low price and high reflectivity. Nonetheless, despite the fact that aluminium has a good corrosion resistance, in certain circumstances its degradation due to this type of problem might be troublesome^3–15,20–23^.

Corrosion of aluminium in solar applications come mainly in the form of two types of corrosion: atmospheric and pitting^23^. Atmospheric corrosion on concentrators can quickly affect their reflectivity, that has a massive impact in the intensity of light that reaches the active area of the solar cell. The pitting scenario leads to errors in the reflection angle, causing non uniformity of light in the receptor. In extreme cases, cells can become shaded or hot spots can be created, promoting serious and unpleasant losses on the system output produced power.

Thereupon, the use of protective treatments and paints to prevent corrosion is seen as essential, the occurrence of corrosion degradation is always unavoidable, mainly because of filiform corrosion, a special case of atmospheric corrosion^24,25^.

Taking into account the aforementioned issues, the aim of this paper is to comprehend how these types of corrosion affect the characteristics of a system composed by an aluminium concentrator and a solar cells, including its power generation.

## Simulations

This software uses a ray tracing algorithm to map the distribution of the irradiance (radiation flux incident on a certain surface: power per area unit) of the concentrator. The ray traicing software code generates randomly, through a Monte Carlo algorithm, rays from the source,“the sun”, and simulates those that are reflected back. The interaction of the overall system with light is a direct consequence of the optical characteristics of the concentrator.

**Figure 3:**
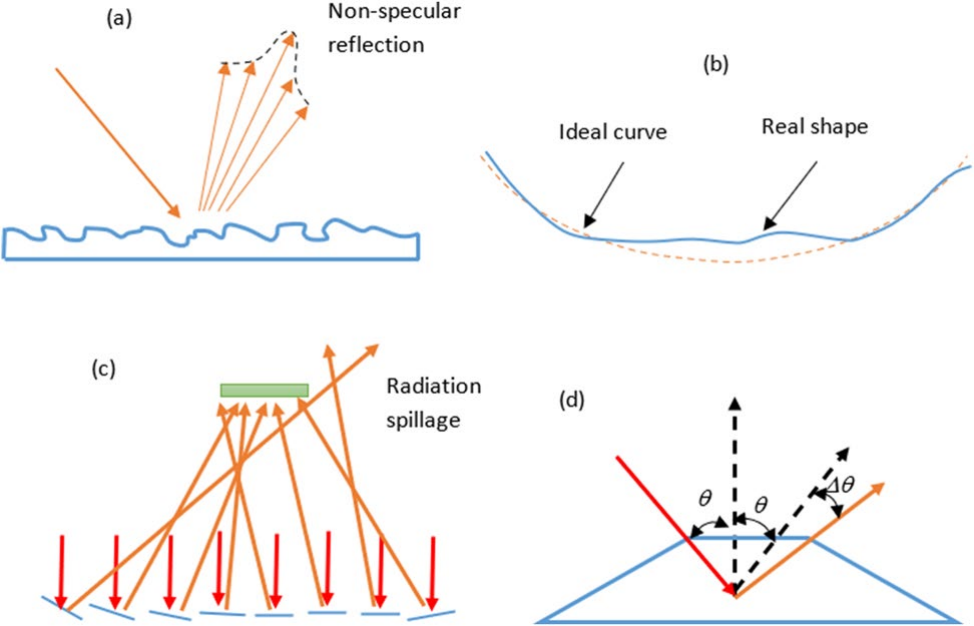
Characteristics Responsible for deviations from the Ideal Reflection (Adapted from^26^).

Typically, a real reflector can be described by two parameters^26^: (i) Average Reflectivity ($R_{avg}$): It is related to roughness of the material that directly affects non-specular reflection, as presented on Fig. 3a; (ii) Standard Deviation ($\sigma _{opt}$): It intends to define the optical error of the surface, according to a Gaussian function, from damages in the service time that result in deviations in the angle of the reflected rays, illustrated on Fig. 3b,c. These parameters allow to analyse the reflective surface not as an ideal one, which is presented on Fig. 3d.

Three reflection-type concentrator shapes are used in the simulation: flat, parabolic and triangular. The last one has twice the area of the flat and their optical parameters are changed in order to acquire the desired conclusions. The receiver is also design to absorb all the radiation.

**Figure 4:**
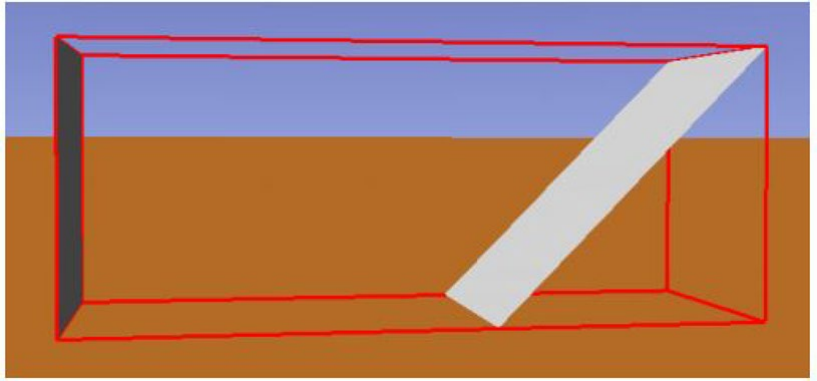
Simulation environment for a flat concentrator: receiver on the left of the flat concentrator.

The flat concentrator has a normalised width of 1 and a normalised height of 1.41. In these simulations, the receiver and the concentrator are placed as illustrated on Fig. 4, having the receiver a unitary area.

**Figure 5:**
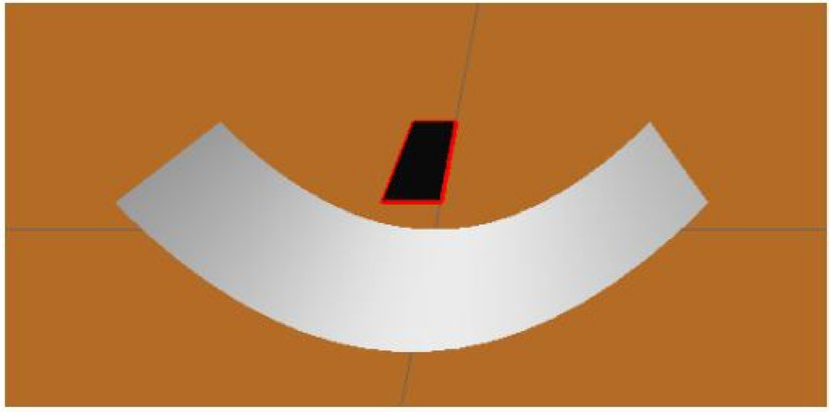
Simulation environment for a parabolic concentrator: receiver on the middle-top of the parabolic concentrator.

On the software, the parabolic concentrator is defined by 5 normalised parameters (lengths): focuslength with a value of 0.25, xMax as the symmetric of xMin, which is also equal to 0.25 and lengthxMin and lengthxMax with unitary value. In these cases, the receiver and the concentrator are placed as illustrated on Fig. 5, having the receiver a unitary area.

**Figure 6:**
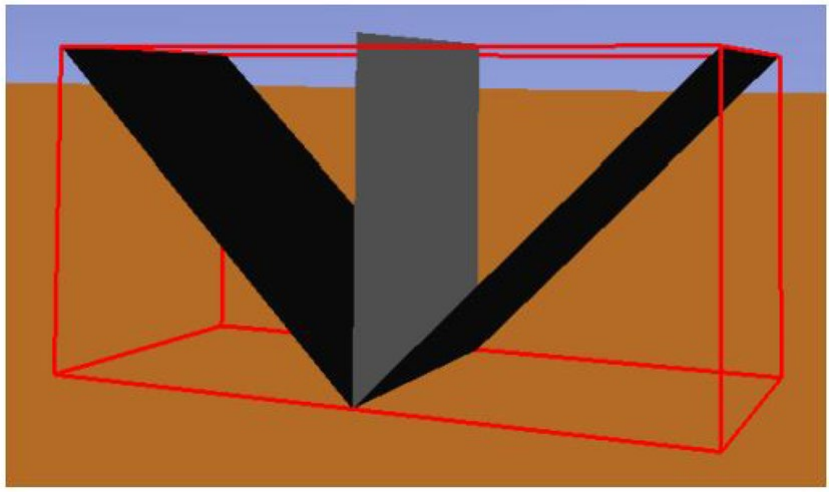
Simulation environment for a triangular concentrator: receiver between two flat concentrators (making a triangular shape).

The last configuration is the triangular one, which is created by two flat plans as illustrated on Fig. 6, such as the triangular shape as twice the flat shape area.

For each simulation, a total of 5 million rays are generated, leading to an irradiance of 1000 W/m$^2$ incident on the concentrator.

### Material reflectivity influence

#### Output power

This simulation is done by changing the reflectivity of the concentrator, measuring the output power for each reflectivity value (in percentage), by determine the irradiance and multiplying its value by the receiver area. This approach assumes the receiver as an absorbing surface, being absorbing all the incident radiation. The reflectivity percentage is determined dividing the reflected rays, on the thin metal surface, by the incident ones. The simulator allows us to use a reflectivity range from 47% (a realistic and trite value) to 100% (very unlikely to reach, with pure materials).

**Figure 7:**
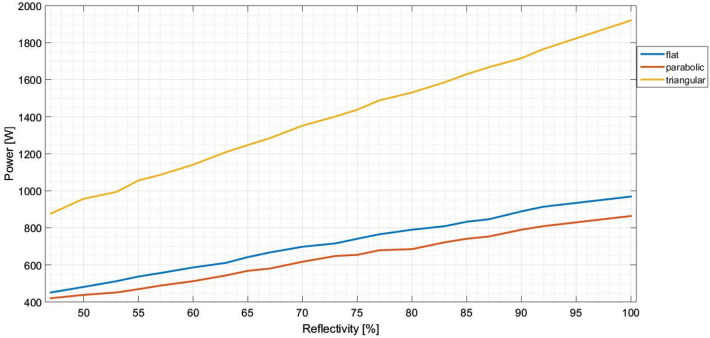
Output power in function of the reflectivity percentage.

The simulation result is presented on Fig. 7, for the three concentrator shapes. Analysing this figure, it is possible to conclude that: (i) The output power has a linear dependence with the reflectivity; (ii) The flat reflector has higher yield than the parabolic one; (iii) There is a linear relationship between the power yielded and the area, because the triangular concentrator (built as two flat concentrators) has twice the output power than the flat one.

However, it is known that in reality, the loss of reflectivity on a curvilinear concentrator is more pronounced, due to the fact that, in its manufacturing process, the defects generated by bending the metal will give rise to areas prone to the creation of pits and/or difficulties in adhesion of the protective paint. In other words, there is a greater probability of corrosion in a folded metal than on a flat one.

#### Flux distribution

**Figure 8:**
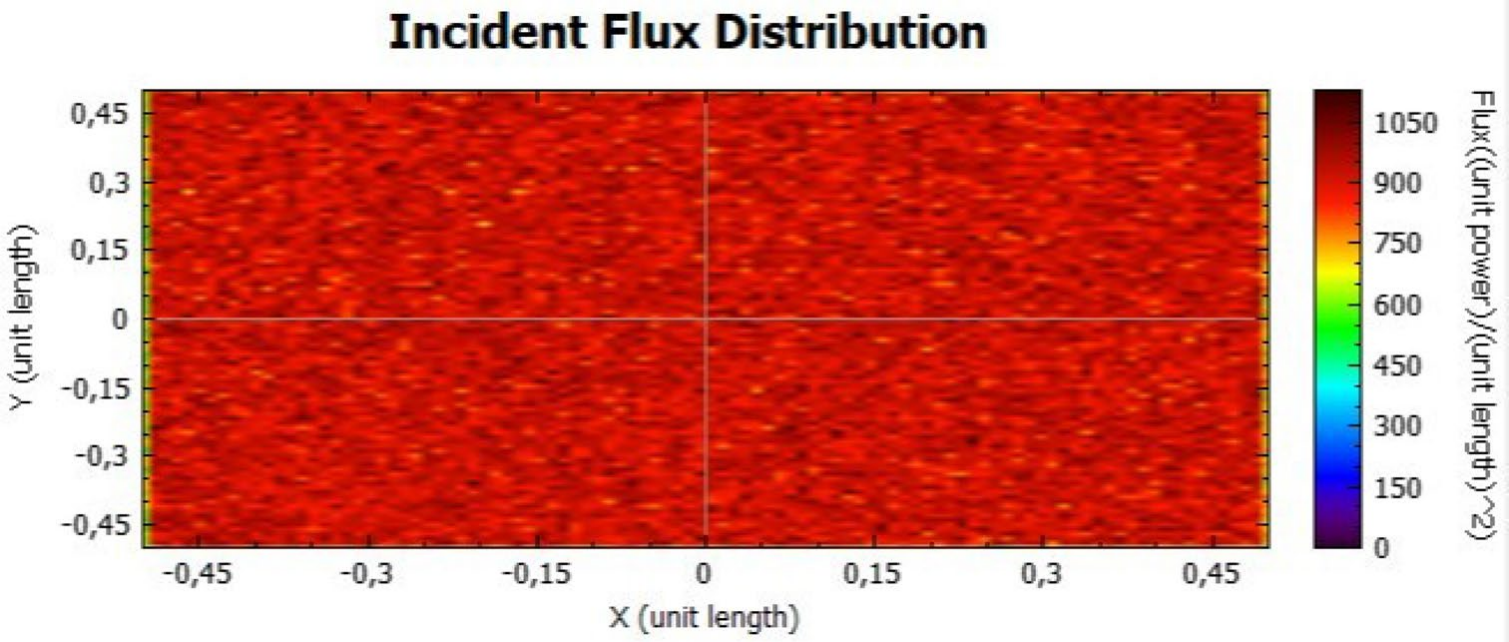
Distribution of incident flux at the receiver from the flat concentrator characterised by a reflectivity of 92%.

**Figure 9:**
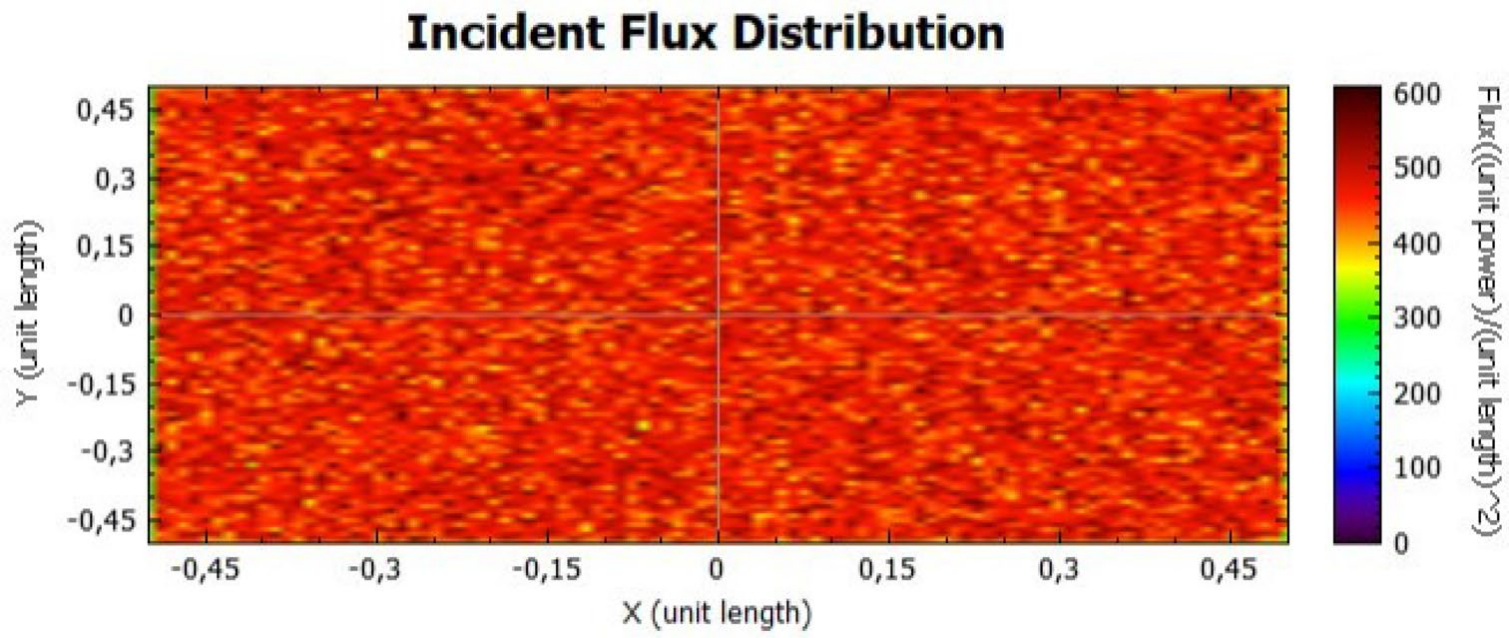
Distribution of incident flux at the receiver from the flat concentrator characterised by a reflectivity of 47%.

In this section, it is analysed the flux distribution on the receiver. In the following figures it is shown the distributions of the incident flux for the flat concentrator with reflectivities of 92% and 47% with null error of standard deviation, presented respectively on Figs. 8 and 9.

When analysing both figures, it is noticeable that the colours are uniform throughout the receiver, indicating, according to the colour scale, that there is no error due to the standard deviation. However, the maximum points on Fig. 8 are higher than the ones on Fig. 9, because the lower the reflectivity is, the lower the power is too.

**Figure 10:**
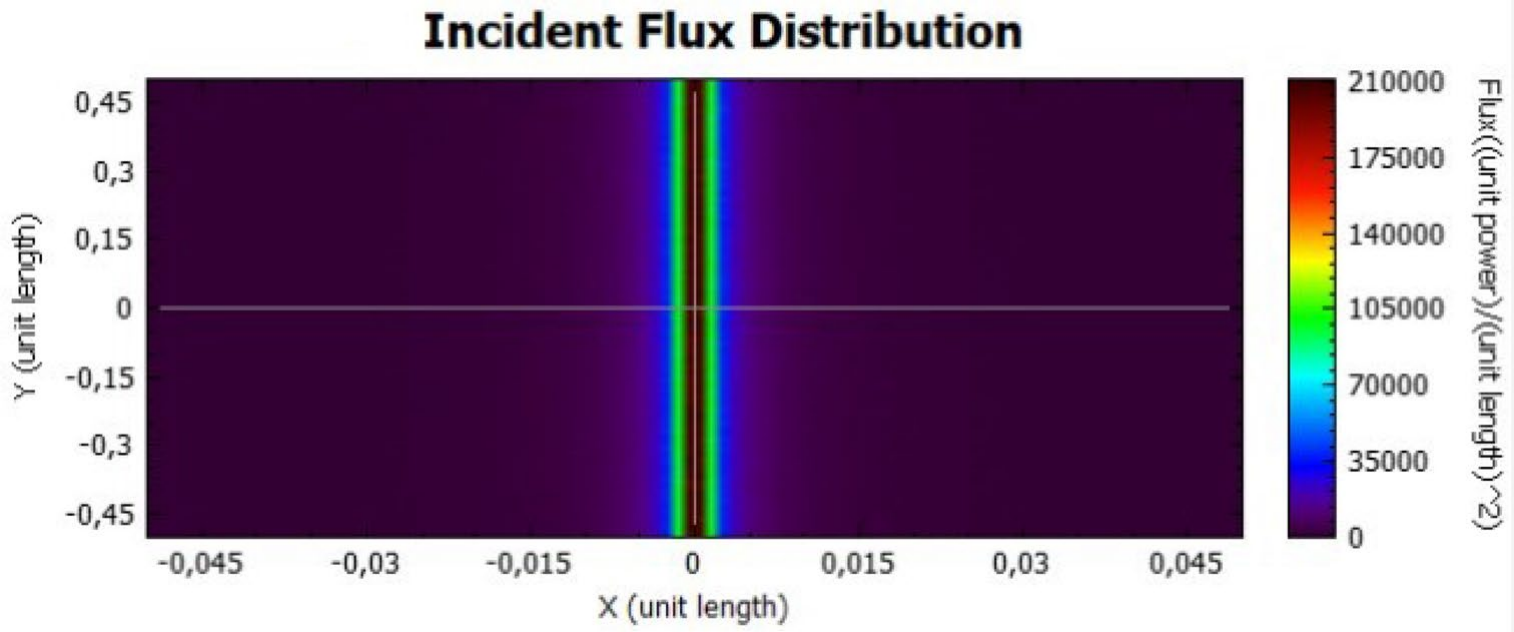
Distribution of incident flux at the receiver from the parabolic concentrator characterised by a reflectivity of 92%.

**Figure 11:**
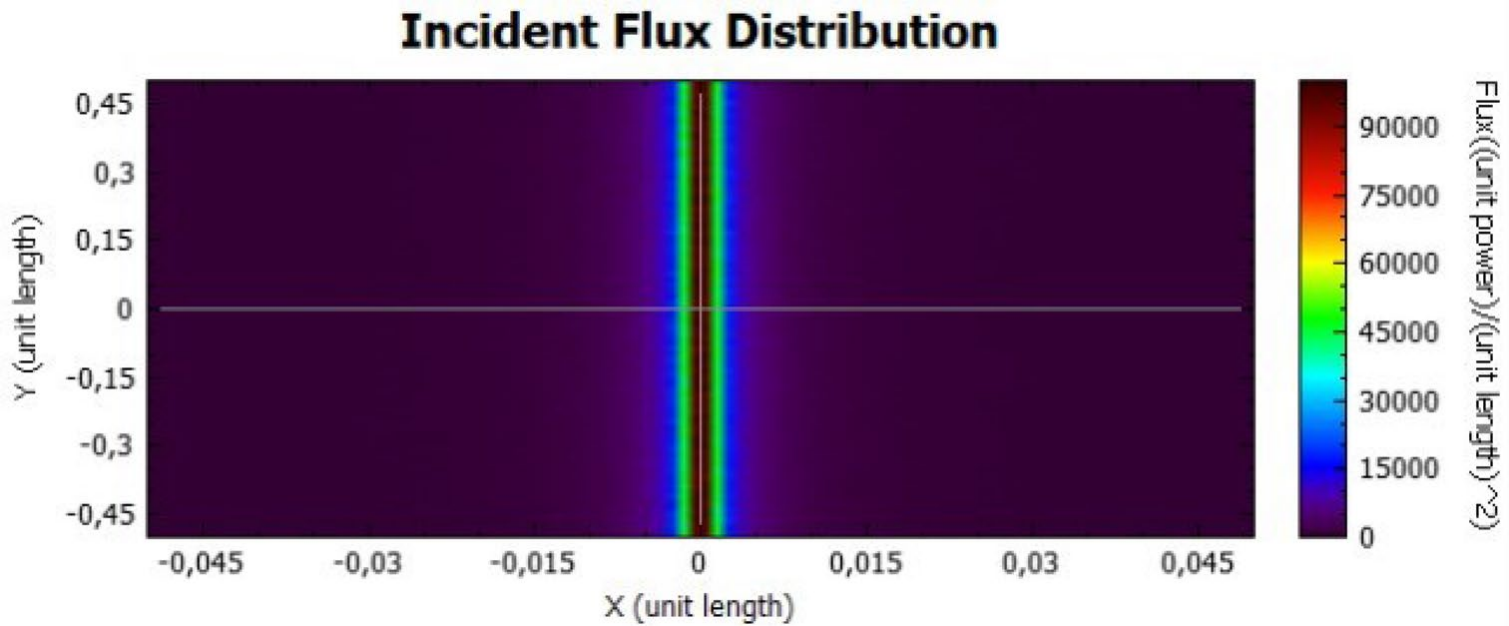
Distribution of incident flux at the receiver from the parabolic concentrator characterised by a reflectivity of 47%.

The parabolic concentrator is also analysed for two different values of reflectivity, as presented on Figs. 10 and 11, for 92% and 47%, respectively. Taking into account the shape of the parabola, the reflected rays will be directed to the central part of the receiver, obtaining high flux values. The flux values are different when both figures are compared, due to the power dependence with the reflectivity.

### Standard deviation influence

#### Output power

**Figure 12:**
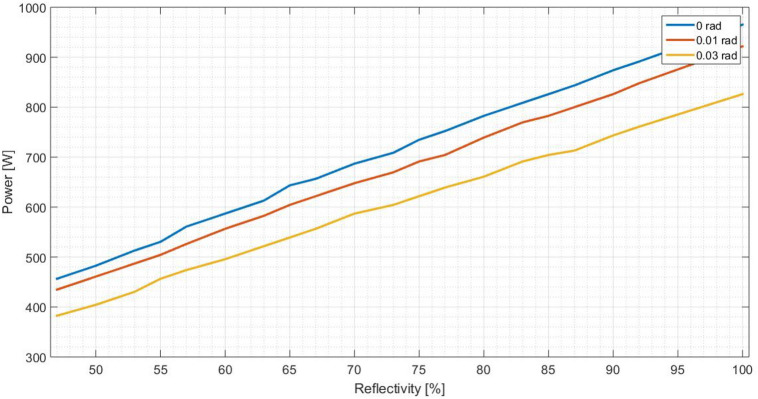
Output power in function of the reflectivity of the flat concentrator, for different optical error values.

**Figure 13:**
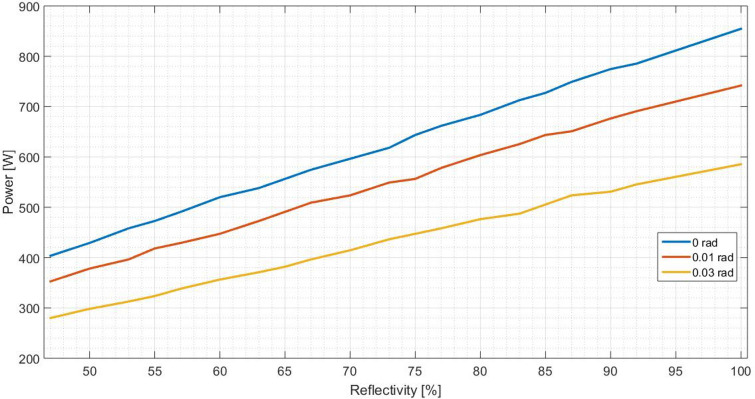
Output power in function of the reflectivity of the parabolic concentrator, for different optical error values.

As previously mentioned, the standard deviation on output power should be evaluated. It is analysed three values associated to the optical error on the surface: 0, 0.010 and 0.030 rad. They are representative and sufficient to obtain the necessary conclusions. For each optical error and reflectivity, the output power are measured. On Fig. 12 it is illustrated the behaviour of a flat concentrator and on Fig. 13 the same is presented for the parabolic one.

Analysing Figs. 12 and  13, it is verified that the higher the standard deviation is, the lower the output power becomes, for both concentrators shapes. In other words, as the error increases, more reflected rays fail the receiver, resulting in a lower received power. Having a rectangular receiver, the worst case is the parabolic concentrator, due to the fact that it is more difficult to position the receiver to catch more radiation, because each ray will have an angle which depends on the incident angle on the concentrator and the punctual parabolic slope.

#### Flux distribution

**Figure 14:**
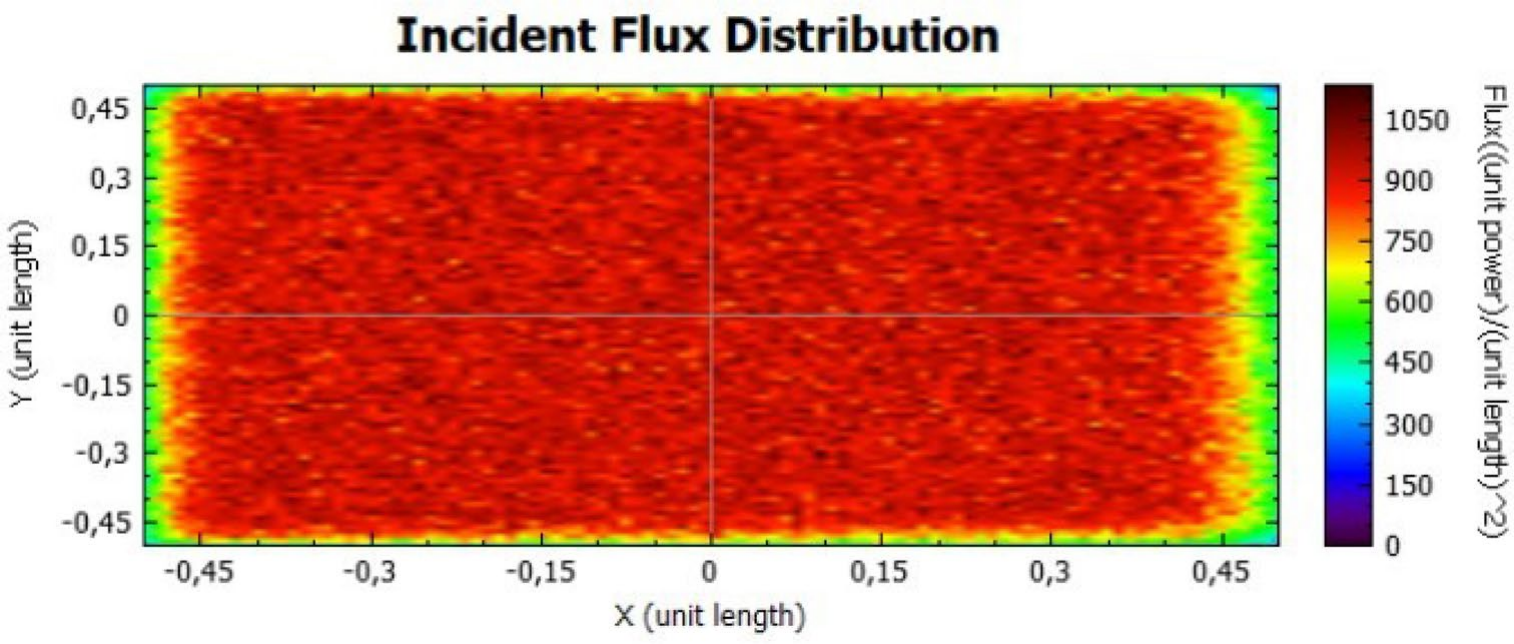
Incident Flux Distribution for an optical error of 0.010 (Flat Concentrator; 92% of reflectivity).

**Figure 15:**
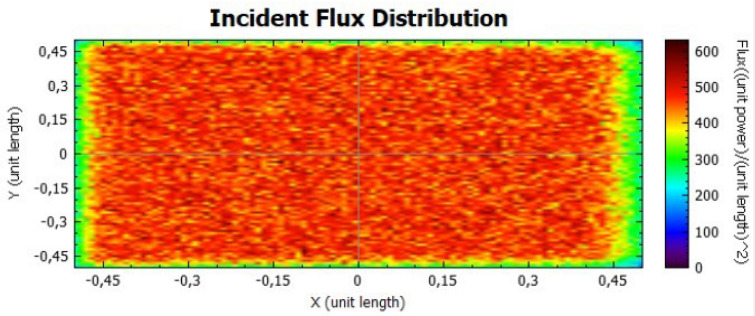
Incident Flux Distribution for an optical error of 0.010 (Flat Concentrator; 47% of reflectivity).

**Figure 16:**
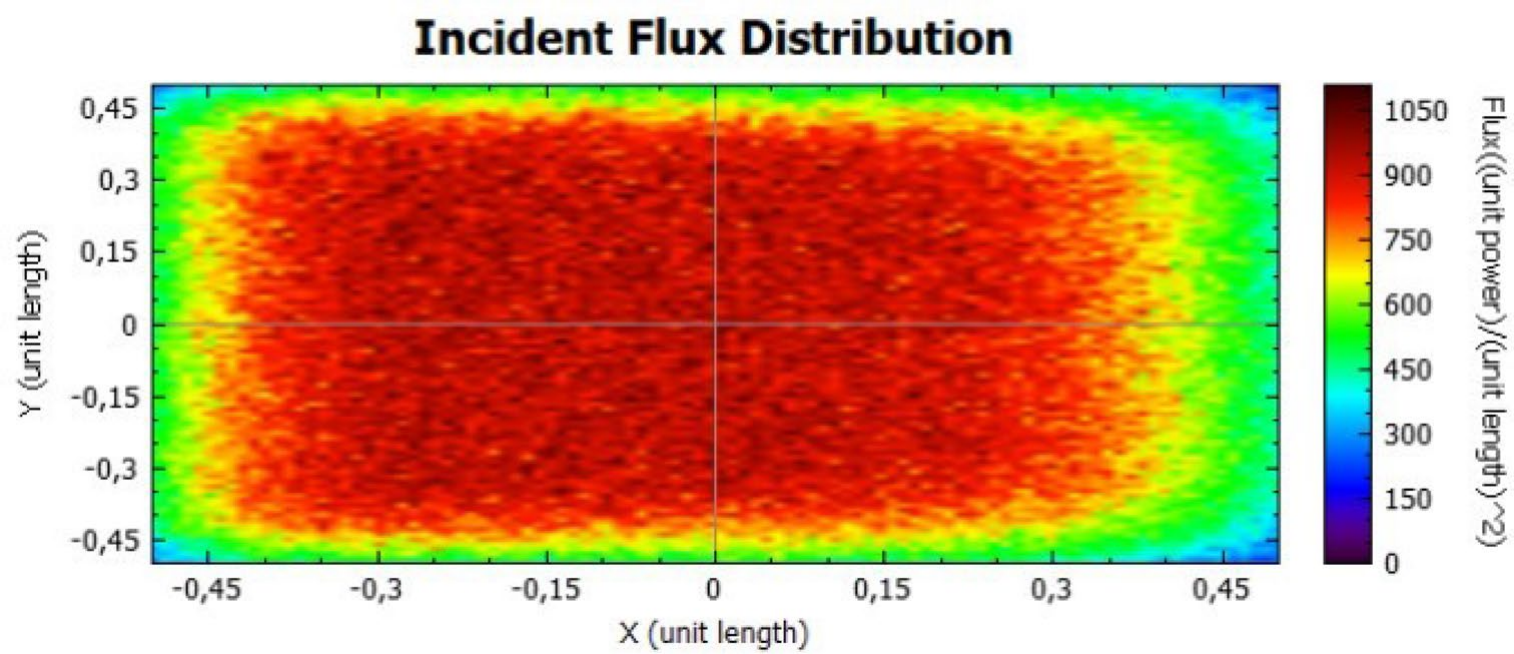
Incident Flux Distribution for an optical error of 0.030 (Flat Concentrator; 92% of reflectivity).

**Figure 17:**
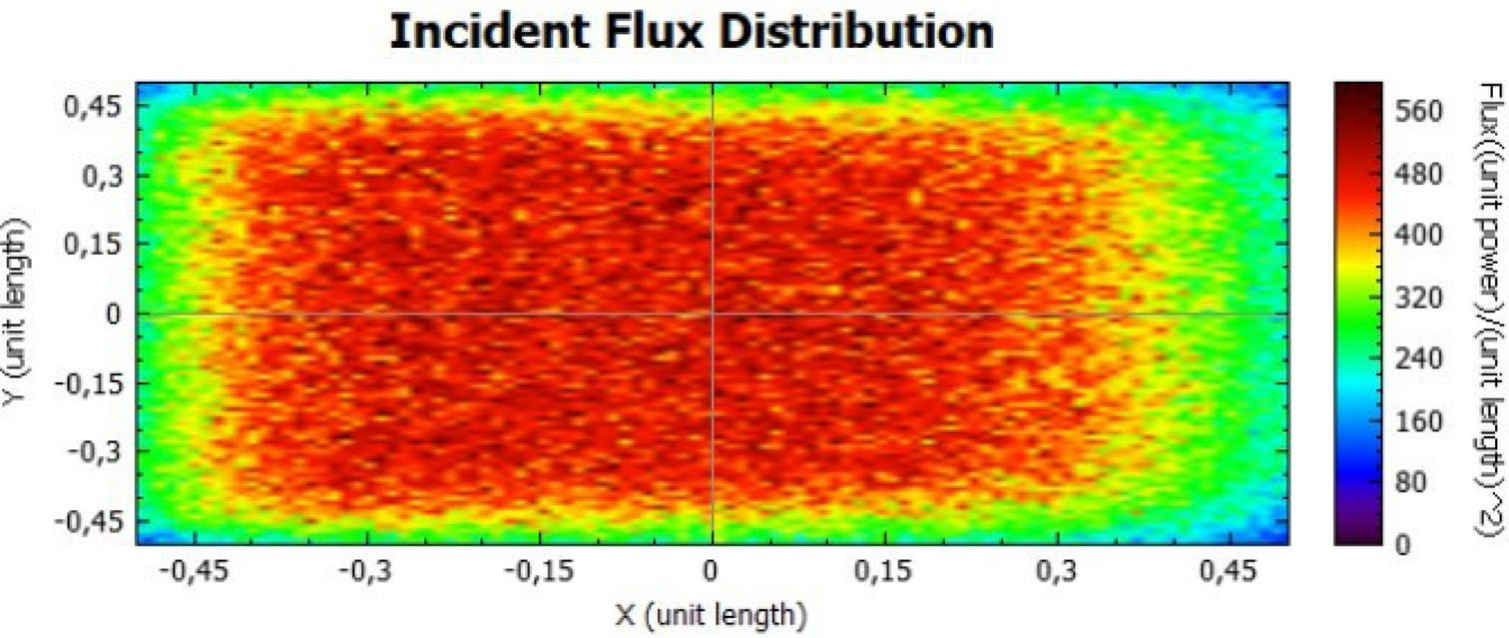
Incident Flux Distribution for an optical error of 0.030 (Flat Concentrator; 47% of reflectivity).

Figures 14, 15, 16 and 17 suggest that there is a diffusion of the incident flux (irradiance) by increasing the standard deviation, observed by the non-uniformity of patterns. In these figures, it is evident the deviation of the expected angle of incidence, illustrated on Fig. 3d. When comparing these figures with Figs. 8 and 9, it is possible to observe a dispersion of the flux leading to a decreasing power reflected.

**Figure 18:**
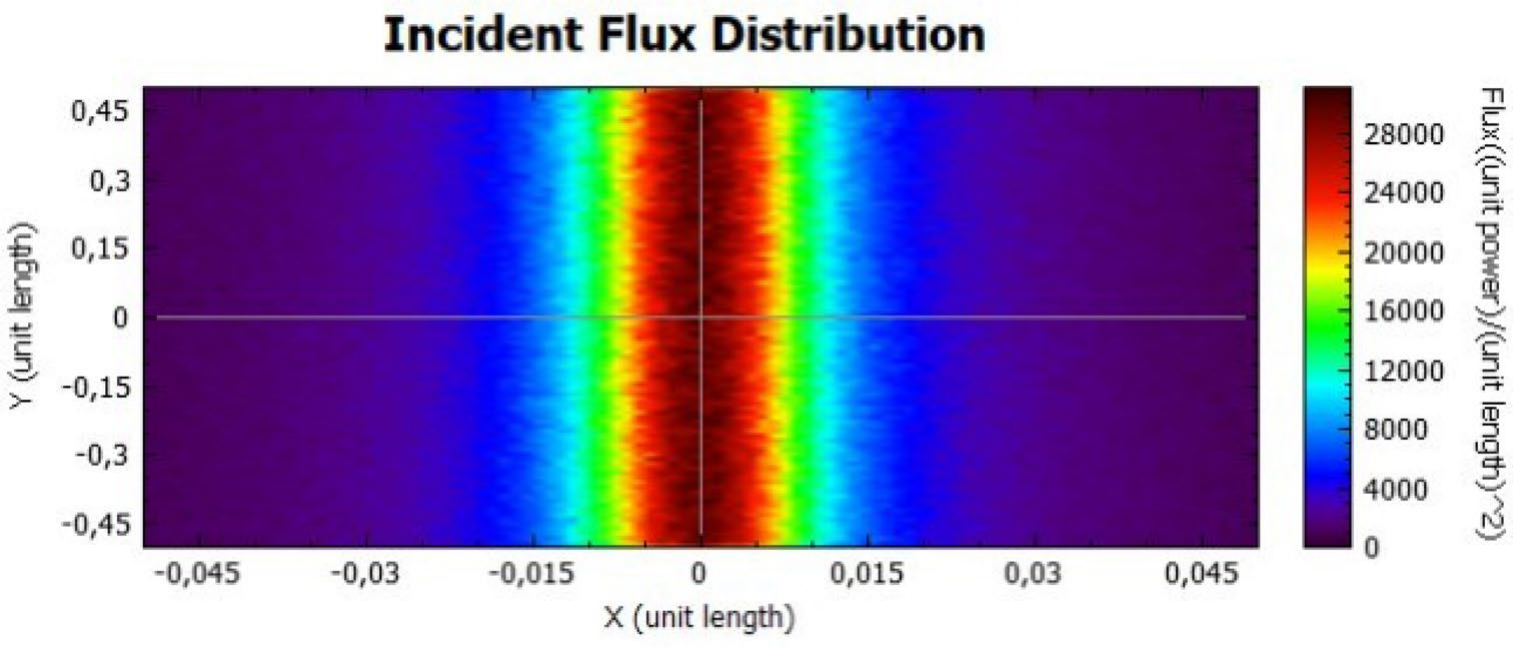
Incident Flux Distribution for an optical error of 0.010 (Parabolic Concentrator; 92% of reflectivity).

**Figure 19:**
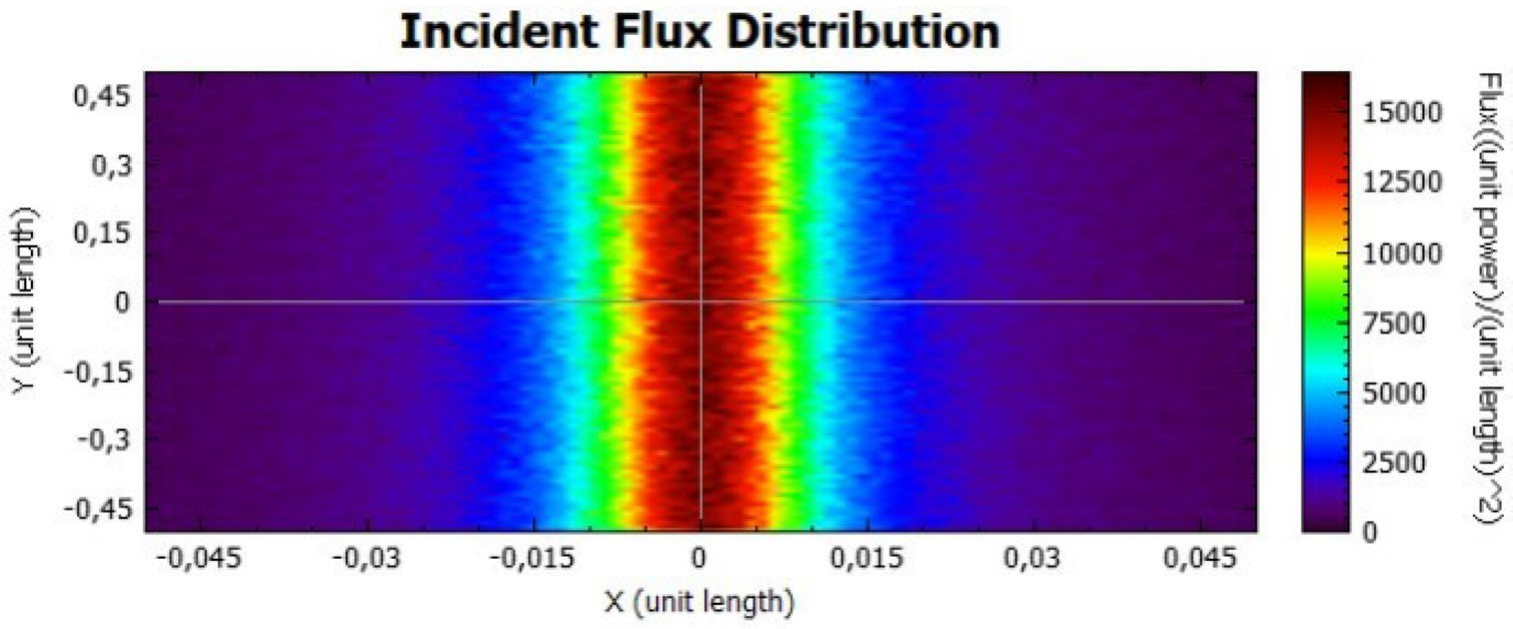
Incident Flux Distribution for an optical error of 0.010 (Parabolic Concentrator; 47% of reflectivity).

**Figure 20:**
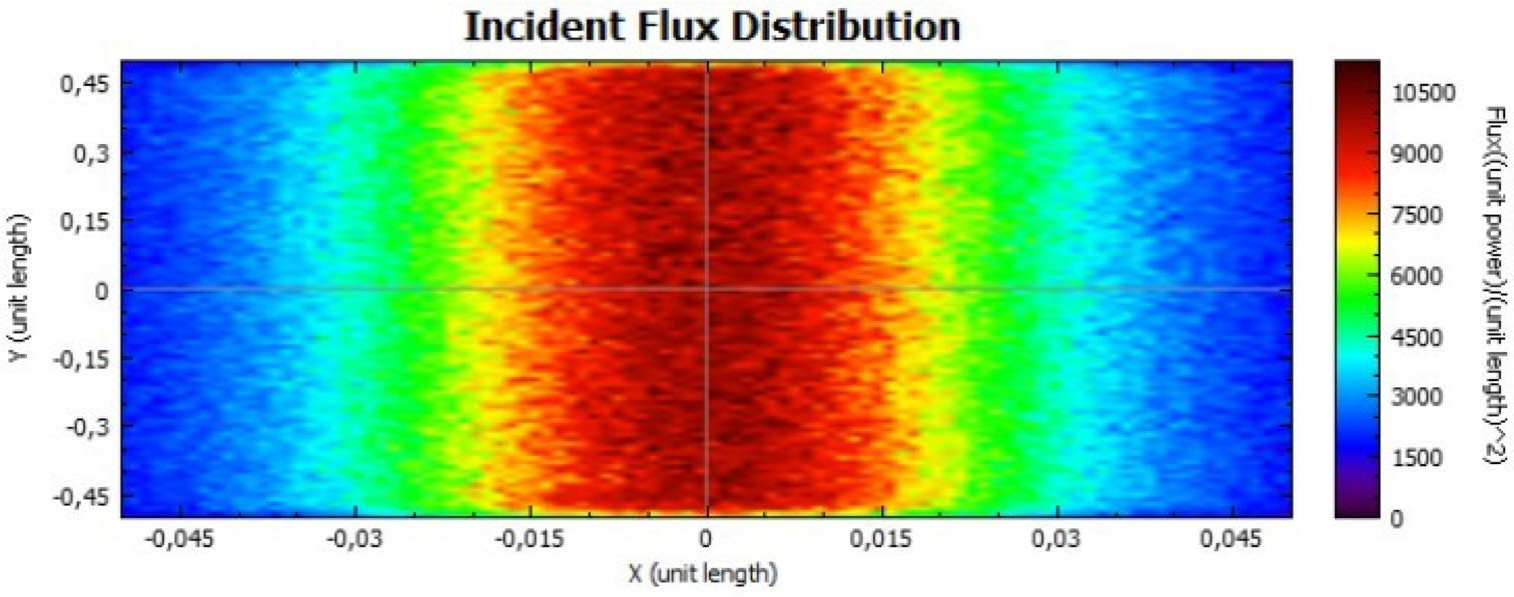
Incident Flux Distribution for an optical error of 0.030 (Parabolic Concentrator; 92% of reflectivity).

**Figure 21:**
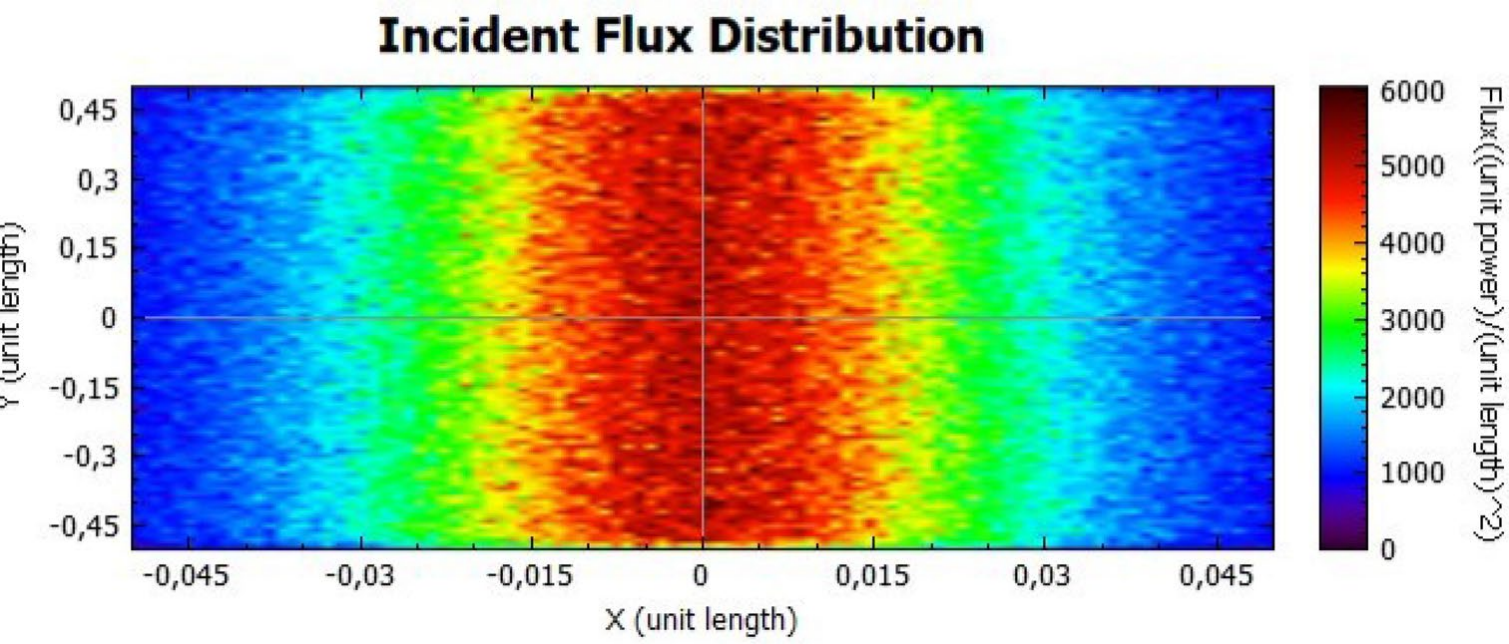
Incident Flux Distribution for an optical error of 0.030 (Parabolic Concentrator; 47% of reflectivity).

In comparison with the flat concentrator, similar conclusion can be taken for the parabolic one, in this case by an analysis of Figs. 18, 19, 20 and 21.

These simulations served to intuitively demonstrate what might happen physically in a solar concentrator and to see how imperfections of the material can change the illuminated zones and consequently, decrease the incident power at the receiver.

Previously, the concepts of short-circuit current, $I_{SC}$, and open circuit voltage, $V_{OC}$, are introduced and it is possible to relate them with these simulations.

Knowing that $V_{OC}$ depends directly on the number of cells connected, it is easy to think that by varying the distribution of the incident power, the number of illuminated cells will change and so that, not only the value of $V_{OC}$ will change too, but all the voltages will vary (for the same load resistance).

On the other hand, the degradation of the concentrator and consequently, the decrease of its reflectivity, changes the intensity with which the photons target the receiver (solar cells), producing a lower photovoltaic current and therefore, a decrease of all the currents values, including $I_{SC}$ (for the same load resistance).

Although the flux distribution and the reflectivity loss are intrinsically associated to the corrosion process, it is possible to approximately split their effects, as it is done on the previous paragraphs.

## Experimental results

It is tested in the laboratory the behaviour of the aluminium plates when submerged in salted water and how this accelerated corrosion process is going to affect the output characteristic of the system composed by the flat aluminium concentrator and silicon solar cells^27^. Four aluminium plates are tested, with different treatments, which are detailed on Table 1.

**Table 1:** Differences between aluminium plates.

Plate number	Colour	Aluminium treatment
1	Red	No treatment
2	Green	No treatment
3	Blue	Polished
4	Light Blue	Painted

### First measurement

First, a test before the immersion in the aqueous solution is done, where the values of $I_{SC}$, $V_{OC}$ and $P_{mp}$ are taken. Based on it, it is possible to verify the differences in the values of reflectivity of the different types of aluminium. When measuring the output current and voltage of the photovoltaic cells, the corresponding power is calculated, and it is conjectured on which aluminium plate had the highest reflectivity value.

This first data, presented on Table 2, suggests that pure aluminium, plates 1 and 2, has a higher reflectivity, followed by plate 3 and 4. Plates 1 and 2 have the same values, because they have not been subjected to any immersion, both being of the same material and without treatment.

Analysing the values of $I_{SC}$ and $V_{OC}$, presented on Table 2, it is possible to infer that the value of current has a greater variation than the voltage one. This is because the current varies directly with the incident illumination current, as previously referred and presented on expressions (6). On expression (5), it is possible to verify that the value of $V_{OC}$ will not vary so much as the value of $I_{SC}$, because it is not a linear dependence, but it will be possible to observe a small variation.

**Table 2:** First measurement of the characteristic values.

Test Number	$I_{SC}$ [A]	$V_{OC}$ [V]	$P_{mp}$ [W]
1	0.100	4.4	0.367
2	0.100	4.4	0.367
3	0.090	4.2	0.307
4	0.081	4.3	0.308

### Evolution of system output characteristics

**Figure 22:**
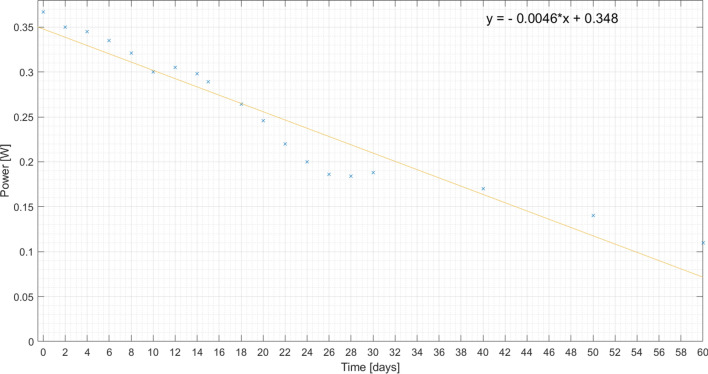
Measured output power on pure aluminium plate (plates 1 and 2) for 60 days and respective linear regression.

**Figure 23:**
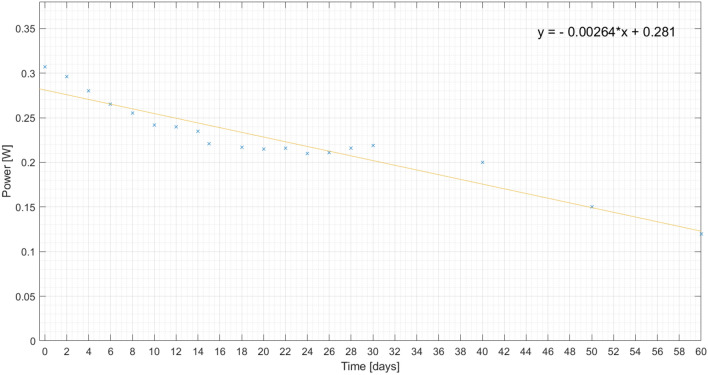
Measured output power on polished aluminium plate (plate 3) for 60 days and respective linear regression.

**Figure 24:**
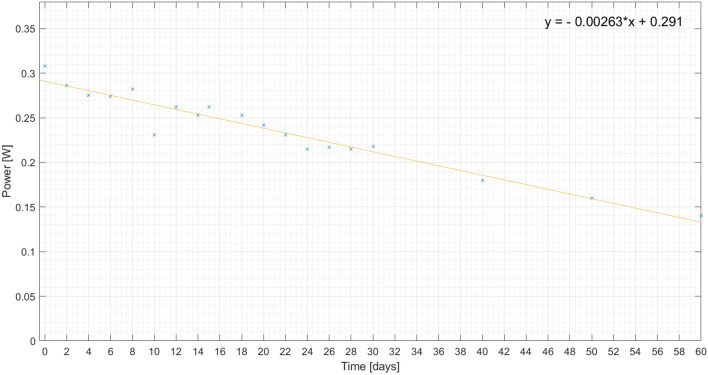
Measured output power on painted aluminium plate (plate 4) for 60 days and respective linear regression.

In order to follow the evolution of the corrosion process, some measurements are made. To confirm that the corrosion progress is different for the plates, the solar cell power is measured and its decrease is observed for 60 days for the same load, as presented on Figs. 22, 23 and 24. Also, a linear regression is performed, in order to get the expression of the power decay along the time interval.

Analysing these figures, it is again confirmed by the slope of the line that the corrosion process is faster in the pure plate of aluminium than in the others.

On Tables 3, 4 and 5 is presented the values of $I_{SC}$, $V_{OC}$ and $P_{mp}$ for each aluminium plates. There, it is possible to observe these values for first measurement, 15 days after it and 30 days after water immersion.

**Table 3:** Evolution of the characteristic values: Pure Aluminium (plates 1 and 2).

Day	$I_{SC}$ [A]	$V_{OC}$ [V]	$P_{mp}$ [W]
0	0.100	4.4	0.367
15	0.084	4.3	0.289
30	0.060	4.1	0.188

**Table 4:** Evolution of the characteristic values: Polished Aluminium (Plate 3).

Day	$I_{SC}$ [A]	$V_{OC}$ [V]	$P_{mp}$ [W]
0	0.090	4.2	0.307
15	0.076	4.2	0.221
30	0.070	3.8	0.219

**Table 5:** Evolution of the characteristic values: Painted Aluminium (Plate 4)

Day	$I_{SC}$ [A]	$V_{OC}$ [V]	$P_{mp}$ [W]
0	0.081	4.3	0.308
15	0.086	4.4	0.262
30	0.075	3.9	0.218

It is noteworthy to verify the linearity with which the maximum power decreases throughout corrosion process. Every 15 days, the power decreases, for pure aluminium, approximately 0.10W and for painted aluminium 0.05W. However, in the case of the polished aluminium plate the degradation seems to be faster at an early stage. This may be because the product used in the polished aluminium has catalysed the oxidation reactions.

In the case of the current there is a decline in all materials, but in the painted plate, this does not manifested in the first 15 days. Furthermore, as previously referred, the $V_{OC}$ is the less susceptible one.

**Figure 25:**
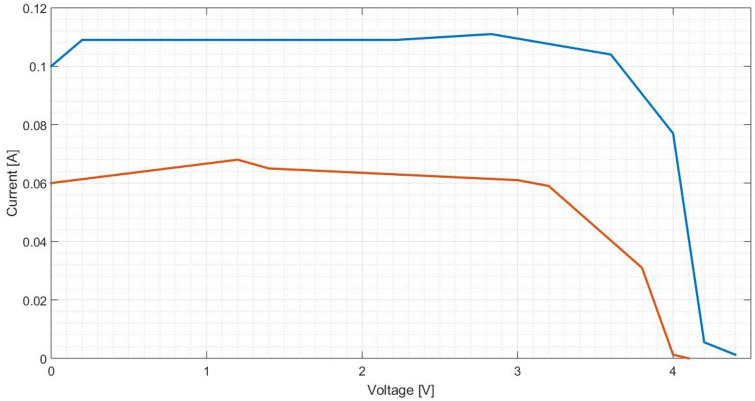
Comparison between I(V) curves of pure aluminium plates (Blue: Day 0; Red: Day 30).

**Figure 26:**
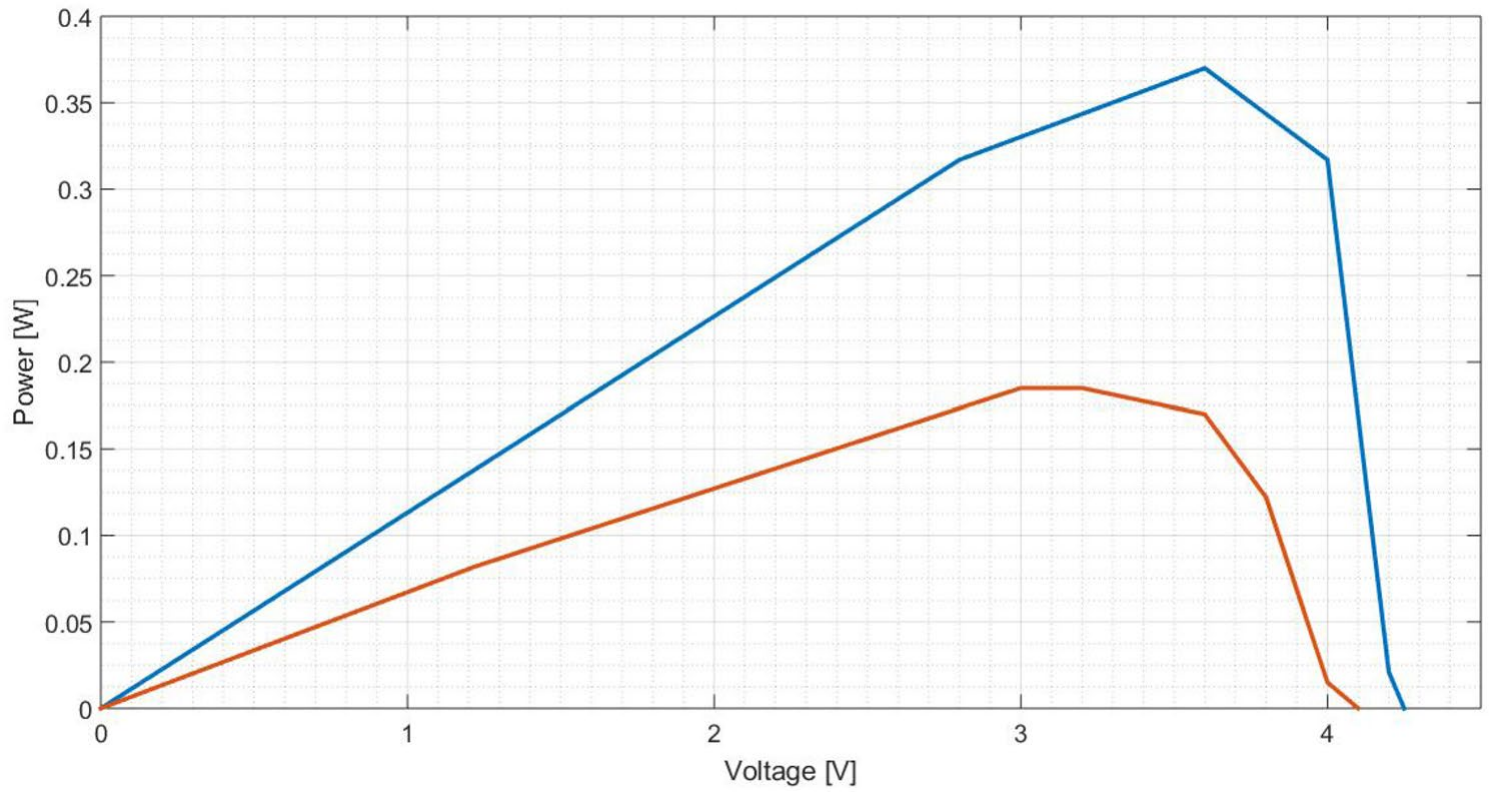
Comparison between P(V) curves of pure aluminium plates (Blue: Day 0; Red: Day 30).

**Figure 27:**
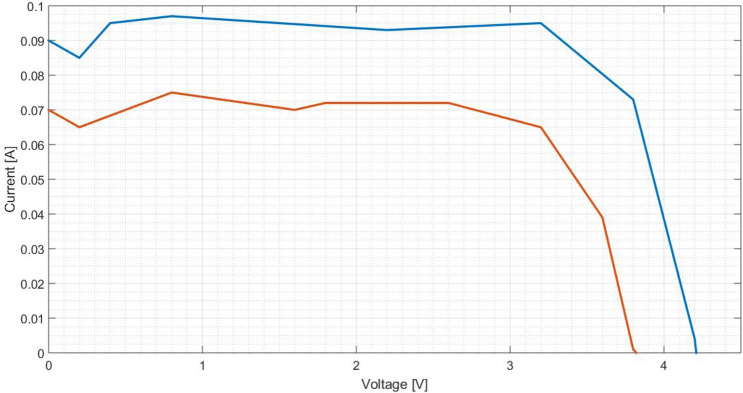
Comparison between I(V) curves of polished aluminium plate (Blue: Day 0; Red: Day 30).

**Figure 28:**
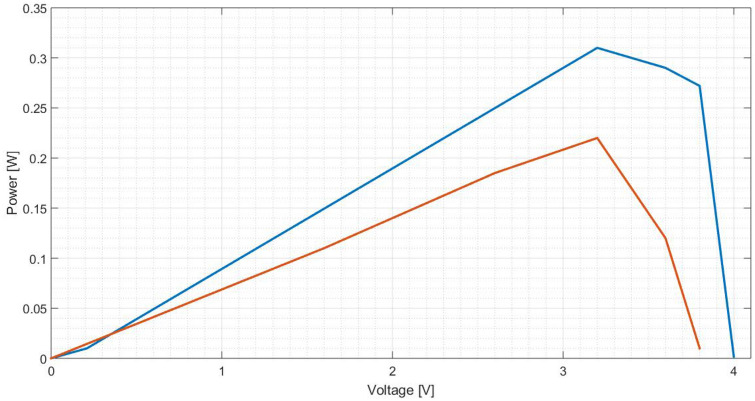
Comparison between P(V) curves of polished aluminium plate (Blue: Day 0; Red: Day 30).

**Figure 29:**
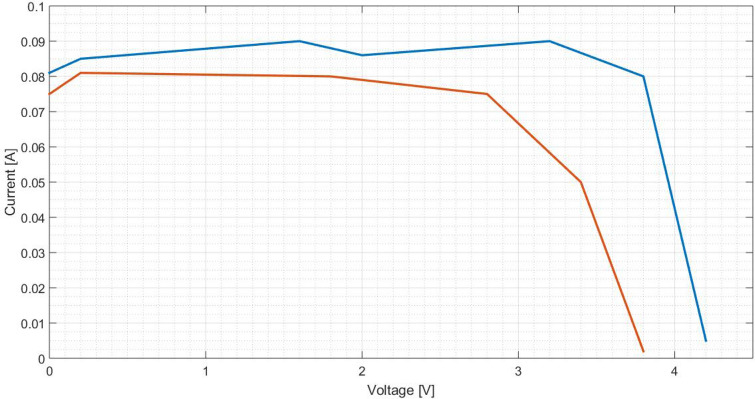
Comparison between I(V) curves of painted aluminium plate (Blue: Day 0; Red: Day 30).

**Figure 30:**
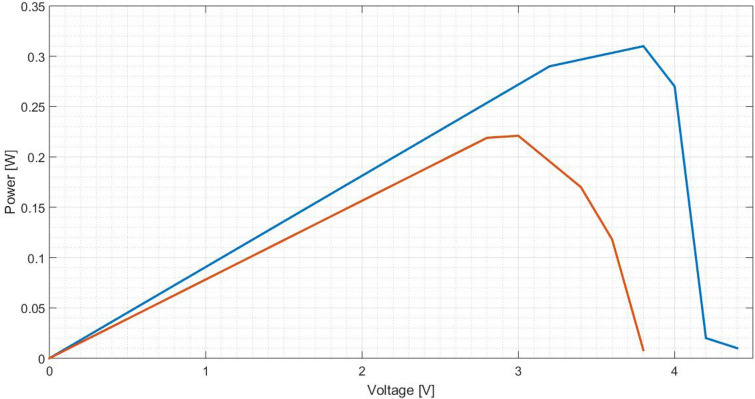
Comparison between P(V) curves of painted aluminium plate (Blue: Day 0; Red: Day 30).

To characterise a solar cell, typically it is presented the curves I(V) and P(V). Thus, other points are taken in order to create these curves. Figures 25 and 26 characterise the cell equipped with a pure aluminium concentrator, Figs. 27 and 28 with the polished one and Fig. 29 and 30 the painted one.

On Table 6 are determined the relative variation of cell characteristics, being the day 0 the reference and the data of day 30 the analysed value.

**Table 6:** Relative variation ($\epsilon$) of characteristic values for the different aluminium plates.

Aluminium plate	$\epsilon _{rel}(I_{SC})$ [%]	$\epsilon _{rel}(V_{OC})$ [%]	$\epsilon _{rel}(P_{mp})$ [%]
Pure	40.00	6.81	48.77
Polished	20.00	9.52	28.66
Painted	12.79	9.30	28.29

Although the comparison is done only considering I(V) and P(V) curves at the beginning and after 1 month (30 days), it is quite obvious that along time both characteristics will tend to zero, based on the power values observed on Fig. 22, 23 and 24, determined along 60 days.

These experimental results are illustrative of the necessity to provide a form to protect the aluminium plate of a concentrator. Even though, as previously verified, a polishing or a painting of the plate leads to an initial decrease of the cell characterisation parameters. However, a treatment allows to maintain a better long-term performance. It is proved because both treated plates exceeded the pure aluminium characterisation parameters and they have lower output power relative variation than the pure one. More over, analysing the linear regression for each plate during the corrosion process, the power slope along time (corrosion velocity) is higher for the pure plate. Thus, depending on the aluminium’s treatment, the time for which the characteristics became null will be different. According to the previous results, it is expected that pure aluminium lead to a null characteristic sooner than polished. In its turn, it is expected that polished aluminium lead to null characteristics before painted aluminium.

## Conclusions

In this article, it is possible to observe how the corrosion of an aluminium concentrator affected the output characteristics of a system composed by it and a silicon solar cells. Some simulations and also laboratory experiments allow us to perform this study.

The first simulation set, using the ray traicing software, allows us to study the variation of the optical parameters of a real concentrator. It is verified that both material reflectivity and the optical error (standard deviation) are two very important variables in order to design a PV system equipped with a concentrator. Their variation influences the incident power on solar cells.

In the first simulation, it is possible to conclude: (1) The decrease in the material reflectivity has a proportional influence on the incident power at the receiver (flatter receiver as more losses than parabolic ones) and does not significantly influence flux distribution, that keeps uniformly distributed on the receiver; (2) The standard deviation (associated to the optical error) modestly modifies the power incident on the receiver and it causes a significant change in the power flux distribution.

After it, experimental results are taken in order to test, in a real system, the influence of the corrosion in a photovoltaic system equipped with a flat aluminium concentrator. The degradation of the concentrator affects the system output characteristics, more specifically its maximum power point, its $V_{OC}$ and its $I_{SC}$. Furthermore, different aluminium plates (untreated, polished and painted) are corroded in containers with salt water and the variation of the cell characteristics is observed. After the experimental period, some conclusions can be pointed out: (1) In the first measurement, the aluminium plate with the highest maximum power is the plate with no treatment, followed by the polished and then the painted one. (2) During the experiments, the plate without treatment had a greater corrosion speed, unlike what happened with the painted plate; (3) After one month, the values for the power using the painted plate are those that had a smaller variation. In the case of using the untreated aluminium, its variation is the largest. Thus, it is suggested that in order to increase the lifetime of the concentrator, the aluminium plates should be treated, being the painted one the one with small relative variations.

The untreated aluminium has more imperfections and a rougher surface, leading to the dispersion of radiation and, consequently, less irradiance will arrive to solar cells. Moreover, since the surface is not polished and it is rough, the corrosion process will be faster, because the material is not treated and it has structure imperfections. The polished plate generates higher power, since it has less structure imperfections and it is characterised by a smoother surface, comparing with the pure aluminium. Thus, light dispersion is, in average, lower, increasing the power efficiency. Further, since the material is treated, the corrosion process will be slower than the pure aluminium one. Considering the painted plate, it is verified that the corrosion process is even slower than for the polished plate. It is the result of the application of a waterproof paint layer that hinders the corrosion process.

These analysis aim to survey the optical interactions between a concentrator and solar cells and thereby promote a development of this interesting technology in terms of cost and efficiency, at a time when it is important to promote and improve renewable energy.
